# Epidermal growth factor receptor-mutant lung cancer in Down syndrome: a case presentation and review of the literature

**DOI:** 10.18632/oncotarget.17406

**Published:** 2017-04-25

**Authors:** Xin Li, Shijiang Xing, Qiumei Dong

**Affiliations:** ^1^ Department of Medical Oncology, Xiaolan People's Hospital Affiliated to Southern Medical University, Zhongshan, China; ^2^ Department of Medical Oncology, Guangdong General Hospital & Guangdong Academy of Medical Sciences, Guangzhou, China

**Keywords:** Down syndrome, non-small-cell lung cancer, EGFR mutation, trisomy 21, EGFR-TKIs resistance

## Abstract

**Background:**

Solid tumors have a markedly decreased incidence in individuals with Down syndrome (DS), including lung cancers.

**Methods:**

The clinical presentation of epidermal growth factor receptor (EGFR)-mutant non-small-cell lung cancer (NSCLC) in DS was reported and literature on the subject reviewed.

**Results:**

In individuals with DS, the risk of lung cancer appears markedly lower. EGFR mutation and EGFR tyrosine kinase inhibitors (EGFR-TKIs) resistance also exist in DS with lung cancer.

**Conclusions:**

Clinicians should consider EGFR mutation and EGFR-TKIs resistance in lung cancer patients with DS.

## INTRODUCTION

Down syndrome (DS) is the most common chromosomal abnormality, which is caused by chromosome 21 trisomy. Approximately 1 in 800-1000 live births are affected, with more than 50% occurring in advanced maternal age pregnancies [1, 2]. While the standardized incidence ratio of cancer in DS was not significantly different from that of the general population, the distribution of malignancies is strikingly different [3]. Increased risk of leukemia in DS is well known [4], but nearly all studies indicate a decreased frequency of solid tumors across all age groups, especially lung cancer, breast cancer, and cervical cancer [5].

Lung cancer, of which NSCLC is the most common form, remains the leading cause of cancer-related mortality worldwide [6]. Treatment NSCLC harbouring mutant epidermal growth factor receptor (EGFR) with specific tyrosine kinase inhibitor (TKI) has led to remarkable tumor shrinkage and improvement in progression-free survival (PFS) and quality of life compared with standard chemotherapy [7–10].

In this report, we describe the rare case of an adult Chinese female with DS who developed an EGFR-mutant lung adenocarcinoma. The management of her cancer is reviewed.

### Case report

A 43-year-old woman with DS was admitted to hospital with a 3-week history of cough, shortness of breath after activities and dyspnea. The patient's motor and sensory status were grossly intact. However, her mental capacity and verbal response to commands were deemed consistent with those of a 5-year-old child. She was a non-smoker and didn't expose to tobacco smoke in her family or known chemical carcinogens for the lung, such as asbestos. And she had no known family history of cancer.

**Figure 1:**
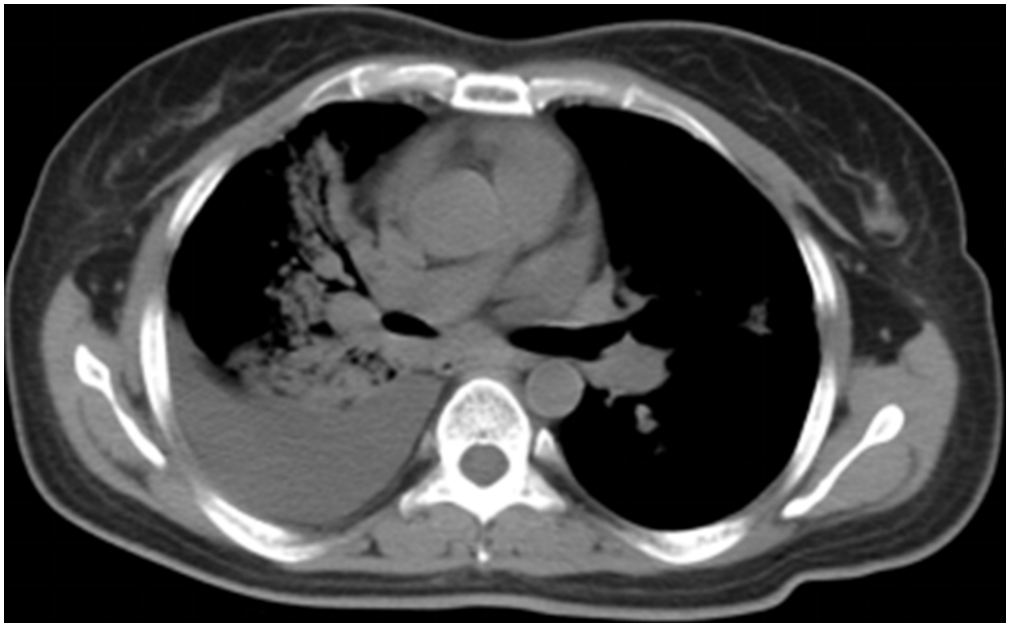
Axial CT image demonstrating pulmonary mass in right central lung, multiple irregular nodules scattered in bilateral lung and right pleural effusion at the time of diagnosis

Clinical examination revealed pulmonary alveolar respiratory sounds were weakened in right lower lung. Pulmonary mass in right central lung, multiple irregular nodules scattered in bilateral lung and right pleural effusion were observed on chest X-ray. Contrast CT scan showed enlarged bilateral mediastinal lymph nodes, metastases of liver segment 4 and eighth thoracic vertebrae, third and forth lumbar vertebrae (Figure 1). Subsequently, the patient underwent CT-guided percutaneous lung biopsy of the right lung mass. After the procedure, the CT scan showed right side hydropneumothorax, but the chest tube drainage did not needed. Pathologic evaluation confirmed the diagnosis of infiltrating lung adenocarcinoma. EGFR mutation status evaluated by allele specific PCR assays (SNaPshot) and PCR-based direct sequencing both showed exon 21 L858R mutation.

**Figure 2:**
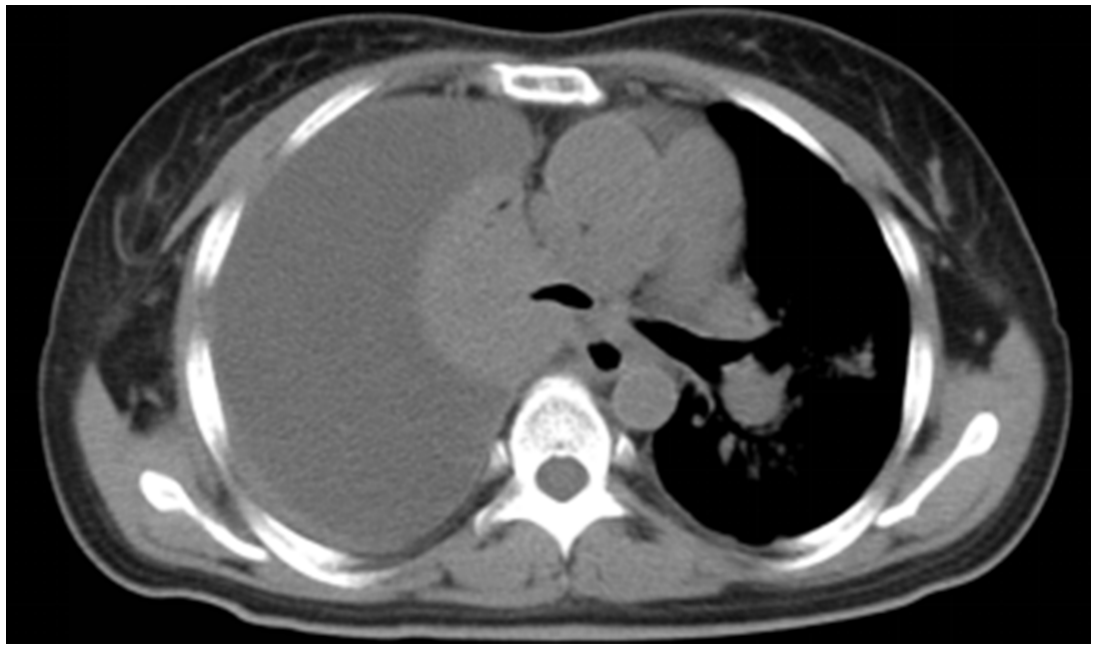
Six months after treated with Gefitinib, a repeat CT scan showed the disease progressed

After a detailed discussion with the family and consent of the mother and sister, the patient was administrated with EGFR TKI- gefitinib at a dose of 250 mg once a day for the first-line treatment. The symptom, such as cough and short of breath both relieved after one week. Two months later, CT scan showed shrinked all of the primary and metastatic tumors and decreased pleural effusion. The principal complications of this regimen were 1 grade diarrhea and rash. A repeat CT scan after six months of therapy was performed due to worsening short of breath. The radiological findings consisted of increase in size of both of the primary tumor as well as liver mass and pleural effusion but not new metastases (Figure 2). The best response of the first-line treatment is stable disease according to Response Evaluation Criteria In Solid Tumors (RECIST version 1.0), and the progression-free survival was six months.

**Figure 3:**
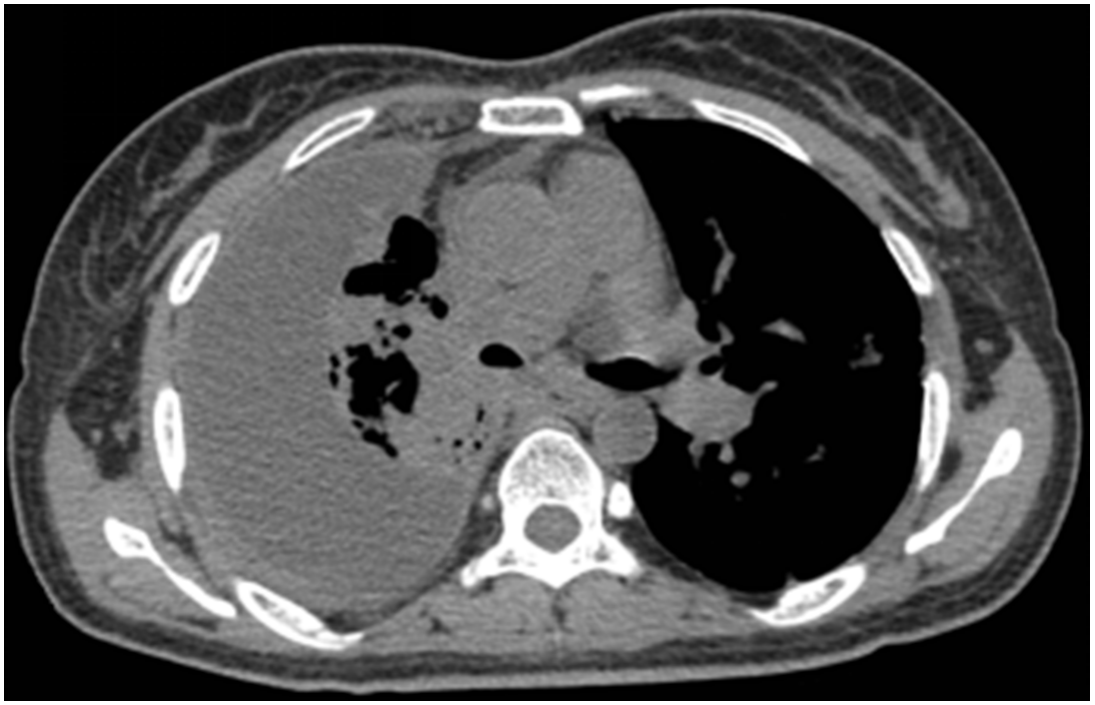
CT scan showed decreased pleural effusion with lung recruitment

We tried to repeat lung biopsy for analyzing mechanisms of acquired EGFR-TKIs resistance, such as T790M, MET amplification or mutations in BRAF, PIK3CA, but her caregivers declined. Following the discovery that T790M is the dominant resistance mechanism to erlotinib and gefitinib [11], therefore the chosen treatment option was the third generation of EGFR inhibitors-osimertinib (at a dose of 80 mg once daily). After one month of second-line treatment, CT scan showed same size all of primary and metastatic tumors and decreased pleural effusion (Figure 3), but new liver metastasis. And short of breath relieved lightly. Unfortunately, two month later she died in respiratory failure at home. No autopsy was performed.

## LITERATURE REVIEW

### Lung cancer risk in patients with DS

DS has been notoriously associated with an increased risk of developing acute leukemia. However, when it comes to malignant solid tumors, they seem to be globally underrepresented with the possible exception of retinoblastoma and germ cell tumors [12].

Lung cancer remains the leading cause of cancer-related mortality in China [13]. It is estimated that 486,555 patients per year died due to lung cancer [13]. But in individuals with DS, the risk of lung carcinoma appears markedly lower across all age groups. Several epidemiological studies on cancer incidence in people with DS, conducted in Denmark, England, Israel, Japan, Finland, Australia [5, 14–18], indicated a reduced risk of lung cancer in this population (Table 1). In these studies, only the Danish study found one lung cancer (in Japanese study benign or malignant is unknown), while 10.0 were expected; the standardized incidence ratio (SIR) was 0.10 (95% CI 0.00–0.56).

**Table 1:** Observed and expected numbers of lung cancer cases and SIR with their CI 95% in patients with DS

	Boker et al^14^	Goldacreet al^15^	Patja et al	Sullivan et al	Hiroaki et al	Hasle et al
Year	2001	2004	2006	2007	2011	2016
Nation	Israel	England	Finland	Australia	Japan	Denmark
Total individuals with DS	1846	1453	3581	1298	1514	3551
All solid tumor cases (N)	13		32	8		55
All solid tumors, SIR		1.2	0.57	0.44		0.45
LC observed			0			1
LC expected			4.2			10.0
LC SIR			0.0			0.10
LC CI95%			0.0-0.9			0.00-0.56

DS: Down syndrome; SIR: standardized incidence ratio; LC: lung cancer.

Satgé et al. [19] reported the first detailed clinical observation of a 33-year-old French man with DS, who developed an adenocarcinoma of the lung 30 years after an acute lymphoblastic leukemia in infancy. But the EGFR-mutation status of this patient was not evaluated due to scarcity of histological material in biopsies. To the authors’ knowledge, this is the first reported case of EGFR-mutant lung cancer in a Chinese patient with DS.

Down syndrome patients are sometimes difficult in understanding, communicating and conveying their symptoms that would have normally helped direct further investigations and identifying underlying tumors. So the diagnoses of malignant tumor sometimes occur too late for an adapted treatment. In this case, when lung cancer was diagnosed, there have been the distant liver and bone metastases. This situation may be improved by greater awareness of symptoms in persons with DS, keeping in mind that cancer may arise.

### Why are lung cancers less common in DS?

At the present time, the mechanism of this decreased risk is not well understood. Several factors have been proposed to contribute to the reduced risk of lung cancers in patients with DS. Environmental factors may play a role, since DS individuals have less likelihood of tobacco and occupational carcinogen exposures, such as asbestos [20]. The gene-dosage effect of the extra chromosome and existence of tumor suppressor gene are also relation to DS.

The frequent occurrence of homozygous deletion and frequent allelic loss which have been repeatedly observed in human NSCLC indicates the presence of a tumor suppressor gene on this chromosome arm [21–23]. Down syndrome candidate region-1 (DSCR1, RCAN1), which encodes a protein that suppresses vascular endothelial growth factor (VEGF)-mediated angiogenic signalling via the calcineurin pathway, is a potential candidate for being a tumor suppressor gene for lung cancer [21–23]. And miRNA genes such as miR-99a, let-7c, and miR-125b-2, have been proposed as possible tumor-suppressive mi-RNAs for lung cancer. However, the role of the other already known genes mapping to chromosome 21 was not completely understood and many other genes remain to be discovered. Future studies should examine the effect of copy number gain and expression of these putative tumor repressor genes on tumorigenesis and cancer progression in individuals without DS. That would help to understand the importance of genetic factor contribution in protecting persons with DS against lung cancer.

### EGFR mutation and EGFR-TKIs resistance with lung cancer

Among patients with advanced NSCLC harbouring mutant EGFR, EGFR-TKIs are the standard first-line therapy [24–27]. Multiple phase 3 clinical trials comparing gefitinib (IPASS, WJTOG3405, NEJ002), erlotinib (OPTIMAL, EURTAC), and afatinib (LUX-Lung 3, LUX-Lung 6) with standard first-line combination chemotherapies have consistently shown a significant improvement in progression-free survival (PFS), objective response rates (ORR), and quality of life for the EGFR-directed therapies [28–34]. EGFR 19 del and 21L858R mutations are the most common sensitive mutations of EGFR, which most commonly found in tumors in women, patients with adenocarcinoma, never or light former smokers, and patients of east-Asian origin [35].

Although EGFR status of the patient Satgé D reported could not be evaluated due to scarcity of histological material in biopsies, we speculate the patient's lung cancer was EGFR-wild type because of the European origin and not respond to the first-generation EGFR-TKI.

Despite high tumor response rates with first-line EGFR-TKIs, disease progresses in a majority of patients after 9 to 13 months of treatment [28–34, 36]. Several resistant mechanisms have been identified, such as T790M missense mutation, amplification of MET, activation of alternative pathways (IGF-1, HGF, PI3CA, AXL), transformation to small-cell histology and epithelial-to-mesenchymal transition [37].

T790M is seen in 50% to 60% of patients with acquired resistance to erlotinib and gefitinib [38]. Many third-generation EGFR inhibitors are in development and have shown excellent activity among patients with T790M-mediated resistance. Recently, a randomized, international, open-label, phase 3 trial (AURA3), comparing Osimertinib with platinum therapy plus pemetrexed in patients with T790M-positive advanced NSCLC (including those with CNS metastases) in whom disease had progressed during first-line EGFR-TKI therapy, have shown a significant improvement in progression-free survival (PFS), objective response rates (ORR) [39].

For this patient whose disease progressed six months after administrated gefitinib, repeated biopsies are now the standard of care [25]. However, the patient's family declined, so the resistance mechanism was unknown. Fortunately, after treated with osimertinib targeting T790M-mediated resistance, the patient have clinical benefits (stable primary and metastatic decreased pleural effusion). But a new liver metastasis was found which should be resistant to osimertinib. Other resistance mechanism may exist in the new metastatic site. Multiple resistance mechanisms at distinct metastatic sites within one patient in this case illustrated the intrinsic heterogeneity of a resistant cancer. C797S mutation located within the tyrosine kinase domain of EGFR was reported to be a leading mechanism of resistance to the third generation irreversible EGFR inhibitors targeting T790M mutation [40, 41]. EAI045 is so far the first allosteric TKI purposefully engineered to overcome T790M and C797S mutations. However it is ineffective alone due to receptor dimerization [42]. But combination with cetuximab renders EAI045 fully active against T790M and C797S [42]. The clinical efficacy of this compound remains unknown at the moment.

## CONCLUSION

This case is exceptional, given the rarity of EGFR-mutant lung cancers in individuals with DS. It is possible that in addition to environmental factors, other factors such as gene-dosage effect and tumor suppressor gene are involved. EGFR mutation and EGFR-TKI resistance also exist in lung cancer with DS. More case reporting should help us gain yet a deeper insight into such a profile.
